# Depressive symptoms, conduct problems and alcohol use from age 13 to 19 in Norway: evidence from the MyLife longitudinal study

**DOI:** 10.1186/s13034-024-00824-x

**Published:** 2024-10-09

**Authors:** Geir Scott Brunborg, Lasse Bang, Jens Christoffer Skogen, Jasmina Burdzovic Andreas

**Affiliations:** 1 Department of Child Health and Development, Norwegian Institute of Public Health, PO Box 222-Skøyen, Oslo, 0213 Norway; 2 Department of Clinical Neuroscience, Karolinska Institutet, Stockholm, Sweden; 3 Department of Health Promotion, Norwegian Institute of Public Health, Bergen, Norway; 4 Centre for Evaluation of Public Health Measures, Norwegian Institute of Public Health, Oslo, Norway; 5 Alcohol and Drug Research Western Norway, Stavanger University Hospital, Stavanger, Norway; 6 Department of Alcohol, Tobacco and Drugs, Norwegian Institute of Public Health, Oslo, Norway; 7 Department of Psychology, University of Oslo, Oslo, Norway

**Keywords:** Adolescence, Depression, Conduct problems, Alcohol, Longitudinal

## Abstract

**Background:**

Even though mental health problems and alcohol use remain major challenges facing adolescents, our understanding of their developmental progressions primarily stems from cohorts coming of age in the early 2000’s. We aimed to examine and describe normative developmental trajectories of depression, conduct problems, and alcohol use across adolescent years among more recent cohorts of Norwegian youth born in the 21st century.

**Methods:**

Multilevel mixed linear models for symptoms of depression and conduct disorder, and multilevel mixed logistic models for depressive disorder, conduct problems, any alcohol use, and risky drinking, were estimated with longitudinal data from a nationwide sample *N* = 3436 (55% girls) of Norwegian adolescents (mean age 14.3 [SD = 0.85] in 2017). We compared models with linear, quadratic, and cubic change with age, and models that tested moderation by sex and centrality (rural vs. urban communities).

**Results:**

Average symptoms and the rate of depressive disorder increased sharply from age 13 to age 19, but both the initial levels and the rates of change were greater for girls than for boys. Average symptoms of conduct disorder and the rate of conduct problems increased in early adolescence and were greater for boys than girls. The rates of any alcohol use and risky drinking both increased sharply from age 14, but there were no notable sex differences either in the initial levels or rates of change over time. Adolescents from more rural communities had greater rates of any drinking in mid-adolescence, but there were no other effects of centrality.

**Conclusions:**

This study provides a much-needed update concerning normative developmental trajectories of depression, conduct problems, and alcohol use among millennium cohorts. Consistent with prior studies, we observed significant increases in all outcomes across adolescence, with depression being both greater and more prevalent among girls and conduct problems being both greater and more prevalent among boys. Consistent with the emerging evidence, we observed no sex differences in alcohol use. Finally, there were no differences in the examined developmental trajectories as a function of centrality. These findings underscore the importance of early prevention and treatment of mental health and substance use problems.

**Supplementary Information:**

The online version contains supplementary material available at 10.1186/s13034-024-00824-x.

## Introduction

Depression, conduct problems, and risky alcohol use are among the most important problems facing adolescents, and as such remain the focus of varied prevention strategies [1–5]. Specifically, increases in depressive symptoms and in rates of depressive disorder are almost normatively observed during adolescence (e.g., 6, 7–9). Changes in conduct problems such as aggression, rule-breaking, and delinquency during adolescence vary with the types of conduct problems, but a general increase in rule-breaking is also typically observed during adolescence [6–9]. Research from several Western countries indicates that there is also a rapid increase in alcohol use and risky drinking starting in mid-adolescence, and that alcohol use does not appear to stabilise or decrease until early adulthood [10–14].

However, with a notable exception of a handful of cohorts born in the early 2000’s [15, 16], our understanding of developmental progressions of these problems remains limited by paucity of robust longitudinal research past youth cohorts born in the 1980s and 1990s. For example, relatively recent reviews and meta-analyses of studies examining developmental trajectories of depressive symptoms among adolescent community samples summarized studies published up until 2015, that is, of pre-2000 cohorts [17]. Similarly, a recent review of studies on adolescent trajectories of conduct problems and alcohol use identified only 13 conceptually and methodologically sound longitudinal studies, and all of them again examined pre-2000 cohorts [5].

Yet, there is emerging evidence that there may be substantive changes taking place when it comes to the symptoms of depression and conduct problems, as well as in alcohol use among young people since the turn of the century. Several studies documented increasing self-reported symptoms of anxiety and depression among adolescents from high-income countries in the last 20 years [18–23]. In contrast, there is evidence of decreasing conduct problems [24–26], whereas decreasing alcohol use among youth across Western societies has been well-documented and already acknowledged as a secular phenomenon [27–30]. Whether these trends are reflecting normative developmental trajectories of depression, conduct problems, and alcohol use during adolescence remains uncertain because, to our knowledge, only a handful of studies have systematically investigated such developments in more recent, post-millennial cohorts [15, 16].

Further, whether there are differences in developmental trajectories of depression, conduct problems, and alcohol use across subgroups of adolescents is not known, despite the relevance of such knowledge for targeted prevention and intervention strategies. For example, the increase in average level symptoms of depression appears to be more rapid for girls than for boys [31]. Overall, depressive disorders increase more rapidly for girls in adolescence, and become more prevalent for girls at the end of adolescence [32–34]. On the other hand, conduct problems and their clinical manifestations (i.e., conduct disorder and oppositional defiant disorder) seem to increase more rapidly and become more prevalent among boys [9]. Research findings are, however, mixed when it comes to differences in adolescent alcohol use trajectories [35], and the heterogeneity in findings might be due to country specific drinking cultures as well as cohort effects. Recent evidence denotes rapid declines in drinking among adolescents, and narrowing of the sex gap in recent cohorts driven by faster declines in alcohol use among boys [36]. At the same time, a recent review pointed at increasing convergence in the development of alcohol use disorders between boys and girls, driven by faster progression into disorder by adolescent girls [37].

Another policy-relevant question is whether the typical development in mental health and substance use may vary as a function of social demography [38]. In Norway, high centrality (closeness to workplaces and service functions) is associated with higher density of goods and service providers, more variation in work opportunities, and higher rates of tertiary education [39]– factors that may influence the onset and progression of depression, conduct problems, and alcohol use among youth. However, previous international cross-sectional studies have reported mixed results [40–45] whereas longitudinal research addressing the role of centrality/urbanity in youth development is lacking.

Against this backdrop, the main aim of the current study was to estimate and describe normative developmental trajectories for symptoms of depression and conduct disorder, and alcohol use among Norwegian youth from ages 13 to 19 years, and trajectories for the corresponding high-risk outcomes (i.e., depressive disorder, conduct problems, and risky drinking). To this end, we used data from a large-scale nationwide longitudinal cohort of Norwegian adolescents born in the period between 2001 and 2003 who were annually assessed five times between 2017 and 2021 with clinically relevant instruments. The secondary aim of this study was to examine potential differences in these developmental trajectories by sex and geographical centrality.

## Methods

### Data source and sampling

The current study used data from the MyLife study. Adolescents from 33 lower secondary schools all over Norway were recruited to ensure a nationwide and geographically and socio-economically heterogeneous sample. Norwegian lower secondary school comprises grades 8 to 10, and nearly all students turn 13 during the year when they start grade 8. Consent, ethical approval and recruitment procedures have been described in detail in the MyLife cohort profile [46]. The project was approved by the Norwegian Data Protection Authority (reference no.: 15/01495) after ethical evaluation by The National Committee for Research Ethics in the Social Sciences and the Humanities (reference no.: 2016/137). Parental consent was required due to the participants’ age. This was obtained for 3512 students that formed a core sample that was invited to complete e-questionnaires at five annual assessments from 2017 to 2021. The analytical sample (*N* = 3436; 55% girls) comprised adolescents who participated at least once in the MyLife study. The number of participants at each timepoint was 2975 (T1), 2857 (T2), 2651 (T3), 2328 (T4), and 1830 (T5). The mean number of assessments for the participants was 3.68 (SD = 1.28). The percentage who missed one, two, three and four assessments were 25.3%, 19.5%, 12.1%, and 7.8% respectively. The mean age was 14.3 years (SD = 0.85) at T1, 15.2 years (SD = 0.84) at T2, 16.2 years (SD = 0.84) at T3, 17.2 years (0.85) at T4, and 18.2 years (0.86) at T5. At T1, 87.6% spoke only Norwegian at home, 10% spoke Norwegian and another language, and 2.4% spoke only another language. One in ten (9.6%) reported experiencing family financial problems.

### Outcome measures

All outcomes were measured at all five time-points.

*Symptoms of depression* were measured with the 9-item Patient Health Questionnaire (PHQ-9 modified for use with adolescents) [47, 48]. The PHQ-9 assesses DSM-IV diagnostic criteria (e.g., low mood, anhedonia, sleep problems, and low energy). Reponses to each item were indicated on 4-point scales where 0 = “not at all” and 3 = “nearly every day”. Detailed examination of the scale properties of the Norwegian version of the PHQ-9 has been presented elsewhere [49]. The sum of the nine item scores was used as a continuous variable in the analyses (scale range was 0–27). Cronbach’s alpha for the scale at the five timepoints ranged from 0.90 to 0.91. Individuals with scores of 15 or higher are likely to meet the diagnostic criteria for Major Depressive Disorder (MDD) with 95% specificity [50, 51]. A dichotomous variable for depressive disorder with the cut-off set at 15 + was also examined in the analyses.

*Conduct problems* were measured using 6 items adopted from the Young in Norway Study [52]. The items assessed symptoms of conduct disorder under each of the core domains in the DSM-5, that is, the frequency of destroying things, fighting, being away at night without parental knowledge, stealing, belligerence, and bullying during the past 12 months. Reponses were made on a 4-point scale ranging from “Never” (coded 0) to “5 or more times” (coded 3). The specific questionnaire items and response frequencies are shown in Supplementary Table 1. The sum of item scores comprised a conduct problems index (range: 0–18) which was used in the analysis. In the DSM-5, the cut-off for conduct disorder is the endorsement of three or more criteria, however because of low cell count, we computed a dichotomous indicator (“conduct problems”) with the cut-off set at 2 + symptoms (i.e., the respondent indicated two or more of the listed conduct disorder symptoms in the last 12 months).

*Alcohol use* was measured with three questions from the Alcohol Use Disorders Identification Test – Consumption (AUDIT-C) [53]: Participants reported drinking frequency in the past 12 months, typical amount consumed per drinking occasion, and frequency of consuming 5 + units of alcohol during a single day. A dichotomous variable for any alcohol use was computed based on the past 12 month drinking frequency item (coded 0 = No alcohol use, 1 = Any alcohol use). The responses to the three AUDIT-C items were scored according to the standards for the AUDIT-C; the scores ranged from 0 to 12. AUDIT-C scores are strongly correlated with alcohol consumption, severity of alcohol problems, and the probability of alcohol use disorders [54, 55]. A conservative cut-off score of ≥ 5 was used to compute a dichotomous risky drinking variable, because this cut-off has been suggested for detecting at-risk drinking and alcohol dependence [56].

### Co-variates

Age in days at each assessment was determined by subtracting each participant’s date of birth from the e-questionnaire submission dates. To anonymize respondents, age in days was transformed to age in years with one decimal for use in the analyses.

The participants’ zip codes were used to identify their municipality’s centrality, according to Statistics Norway’s centrality index [39]. The centrality index ranges from 1 to 1000 and is determined by the number of different service functions and different types of workplaces that residents on average can reach within 90 minutes’ drive from home, adjusted for travel time. Three centrality levels (low, mid- and high centrality) were used in the analysis. The sample distribution was 39.1%, 44.9%, and 16.0% for these levels respectively.

### Analysis

Growth curve modelling within a multilevel modelling framework was used to estimate the development in all outcomes, as described by Singer and Willett [57]. To estimate developmental trajectories in depression, conduct problems, and alcohol use from age 13 to 19, we exploited the sequential cohort design of the MyLife study, and age was used as the time metric rather than assessment years [58]. We fitted two-level models: The first level was age (centered at 13 years) whereas the second level comprised individual participants. For continuous outcomes, we fitted multilevel mixed-effects linear regression with the ‘mixed’ command in Stata 16; for dichotomous outcomes we fitted multilevel mixed-effects logistic regression using the ‘melogit’ command.

The shapes of the developmental trajectories were determined first. For each outcome, we fitted four basic growth models for change with age: intercept only (i.e., no change with age), linear change, quadratic change, and cubic change. Improvement in model fit was assessed with reduction in the deviance statistic [57] and associated χ*²* difference tests. To reduce the risk of overfitting the model to the data, we also considered any reduction in Akaike’s Information Criterion (AIC) and the Bayesian Information Criterion (BIC).

Next, we tested for potential sex and potential centrality moderation by including interaction terms with the growth parameters (e.g., intercept x sex; linear slope x sex; quadratic slope x sex). Reduction in the deviance statistic and in AIC and BIC were used to examine if there was evidence of moderation.

Estimated marginal means based on the best fitting models were obtained by using the ‘margins’ command in Stata. For models with continuous outcomes, we specified the unstructured covariances structure and specified random effects for the intercept and the linear slope. For dichotomous outcomes, we specified random effects for the intercept. The models were estimated with full maximum likelihood. All the available data were used for estimation, and missing outcome values were not imputed [59]. Robust standard errors clustered at schools were used in all analyses to account for the nesting of individuals in schools.

The multilevel regression modelling resulted in a large number of *p*-values. We adjusted the alpha level for statistical significance with the Benjamini–Hochberg procedure [60], based on all the multilevel regression coefficients’ *p*-values, to control the type I error rate.

### Attrition

To examine study attrition, the dichotomous outcome variables measured at T1 (depressive disorder, conduct problems, and risky drinking) as well as sex, age, and centrality were included in four separate logistic regression models where the outcomes were non-participation at T2, T3, T4 and T5 respectively. Older age at T1 predicted non-participation at all the later timepoints (OR = 1.65, 1.54, 1.20 and 1.12 respectively for a one-year increase in age). Male sex predicted non-participation at T3 (OR = 1.44), T4 (OR = 2.04) and T5 (OR = 2.19). Finally, conduct problems predicted non-participation at T4 only, OR = 1.61 (all *p*s < 0.01). Depressive disorder and risky drinking at T1 did not predict non-participation at any of the subsequent timepoints.

## Results

Summary of all studied outcomes from age 13 to 19 separately by sex are shown in Table 1. For girls, the average symptoms of depression increased with each passing year, as did the prevalence (i.e., proportions) of depressive disorder. The observed trend was similar for boys, but the boys’ values were considerably lower overall, and there was a peak at age 18. Both sexes peaked at age 18 with regards to depressive disorder.

**Table 1:** Sample means (SD) and proportions for all study outcomes from age 13 to 19 years, with tests for sex differences

Outcomes	Age						
	13	14	15	16	17	18	19
**Symptoms of depression**							
Girls	5.55 (5.26)	7.42 (6.01)	8.62 (6.06)	9.27 (6.14)	10.15 (6.36)	10.34 (6.09)	10.44 (6.15)
Boys	3.94 (4.24)	4.40 (4.21)	5.06 (4.54)	5.76 (5.06)	6.31 (5.39)	7.13 (5.41)	6.75 (5.22)
*t*	3.960	11.199	16.266	15.598	15.075	10.481	8.308
*p*	< 0.001	< 0.001	< 0.001	< 0.001	< 0.001	< 0.001	< 0.001
Cohen’s *d*	0.33	0.57	0.65	0.62	0.64	0.55	0.63
**Depressive disorder**							
Girls	5.8%	13.1%	16.4%	18.1%	22.6%	23.95%	23.1%
Boys	4.0%	2.8%	4.0%	6.6%	8.1%	11.62%	7.9%
*t*	0.978	51.729	96.068	74.126	82.716	35.591	27.428
*p*	0.323	< 0.001	< 0.001	< 0.001	< 0.001	< 0.001	< 0.001
**Symptoms of conduct disorder**							
Girls	0.27 (0.74)	0.38 (0.99)	0.45 (1.14)	0.46 (1.15)	0.52 (1.27)	0.51 (1.39)	0.43 (1.08)
Boys	0.63 (1.29)	0.81 (1.74)	0.88 (1.99)	0.97 (2.10)	1.07 (2.30)	0.91 (1.93)	0.86 (1.82)
*t*	-4.427	-6.415	-6.929	-8.072	-7.383	-4.725	-4.06
*p*	< 0.001	< 0.001	< 0.001	< 0.001	< 0.001	< 0.001	< 0.001
Cohen’s *d*	-0.35	-0.31	-0.27	-0.31	-0.31	-0.25	-0.31
**Conduct problems**							
Girls	4.4%	7.8%	7.8%	8.0%	8.2%	7.4%	5.7%
Boys	9.6%	14.7%	15.0%	16.6%	15.3%	15.0%	12.6%
*t*	6.909	20.689	34.214	47.666	28.229	22.989	11.044
*p*	0.009	< 0.001	< 0.001	< 0.001	< 0.001	< 0.001	0.001
**Any alcohol use**							
Girls	3.9%	9.2%	26.7%	47.3%	65.9%	78.5%	90.2%
Boys	3.6%	11.5%	23.4%	39.4%	57.5%	73.7%	87.3%
*t*	0.027	2.386	3.637	16.465	16.675	4.814	1.477
*p*	0.869	0.122	0.057	< 0.001	< 0.001	0.028	0.224
**Risky drinking**							
Girls	0.3%	0.8%	3.7%	10.0%	19.7%	34.7%	44.2%
Boys	0.0%	1.5%	4.5%	11.2%	25.3%	35.6%	51.1%
*t*	0.765	1.599	0.943	0.950	10.168	0.134	3.358
*p*	0.382	0.206	0.331	0.330	0.001	0.715	0.067

Age in years with one decimal is rounded to the nearest whole number; SD: Standard deviation

The frequencies shown in Supplementary Table 1 indicate that conduct problems were unusual, but nevertheless reported by some adolescents. The average symptoms of conduct disorder and the prevalence of reporting two or more conduct problems increased in early adolescence but declined after age 17. Symptoms of conduct disorder, and the proportion of the participants with two or more conduct problems, were roughly twice as high for boys compared to girls.

The prevalence of any alcohol use and of risky drinking increased rapidly with age. Although the overall differences between the sexes were small, at age 16 and 17, the rate of any alcohol use was somewhat higher for girls, whereas the risky drinking rate was higher for boys at age 17.

### Comparing growth curve models

The first step of the growth curve modelling was the comparison of the estimated linear, quadratic, and cubic growth models for the six study outcomes (see Table 2). For all outcomes, the model fit improved after adding a linear slope term, and it improved further by adding a quadratic slope term, as indicated by decreases in the deviance static, AIC and BIC. For none of the outcomes did the model fit improve after adding a cubic slope term.

**Table 2:** Comparison of the linear, quadratic, and cubic growth curve models

Model	Pseudo log likelihood	Deviance	df	∆χ²	∆df	*p*	AIC	BIC
**Symptoms of depression**								
Intercept only	-37,137	74,275	3				74,281	74,303
Linear change	-36,665	73,330	6	945	3	< 0.001	73,342	73,387
Quadratic change*	-36,623	73,245	7	85	1	< 0.001	73,259	73,311
Cubic change	-36,623	73,245	8	0	1	0.581	73,261	73,320
**Depressive disorder**								
Intercept only	-4250	8500					8504	8518
Linear change	-4148	8296	3	203	3	< 0.001	8302	8324
Quadratic change*	-4139	8277	4	19	1	< 0.001	8285	8315
Cubic change	-4138	8277	5	1	1	0.417	8287	8324
**Symptoms of conduct disorder**								
Intercept only	-22,706	45,412	3				45,418	45,440
Linear change	-22,610	45,220	6	192	3	< 0.001	45,232	45,277
Quadratic change*	-22,603	45,206	7	14	1	< 0.001	45,220	45,272
Cubic change	-22,603	45,206	8	0	1	1.000	45,222	45,281
**Conduct problems**								
Intercept only	-3791	7581	2				7585	7600
Linear change	-3789	7579	3	3	1	0.108	7585	7607
Quadratic change*	-3781	7562	4	17	1	< 0.001	7570	7600
Cubic change	-3779	7558	5	4	1	0.047	7568	7605
**Any alcohol use**								
Intercept only	-8158	16,316	2				16,320	16,335
Linear change	-5576	11,153	3	5164	1	< 0.001	11,159	11,181
Quadratic change*	-5567	11,135	4	18	1	< 0.001	11,143	11,172
Cubic change	-5566	11,132	5	3	1	0.094	11,142	11,179
**Risky drinking**								
Intercept only	-5135	10,270	2				10,274	10,289
Linear change	-3896	7792	3	2478	1	< 0.001	7798	7821
Quadratic change*	-3880	7759	4	33	1	< 0.001	7767	7797
Cubic change	-3880	7759	5	0	1	0.572	7769	7806

*Model selected

The second step was to test moderation, that is, we examined if specifying different growth curves for boys and girls would improve model fit, and secondly, if specifying different growth curves for the three centrality levels would improve model fit (see Table 3). For all outcomes except the two alcohol outcomes, the model fit was superior for the models that specified different growth curves for girls and boys. The models that specified different growth curves for low, mid-, and high centrality fitted the data more poorly than the models with no moderation. For any alcohol use, the model that specified different growth curves for the three centrality categories was the best fitting model. However, for the risky drinking outcome, the model that specified no moderation was the best fitting model.

**Table 3:** Testing moderation by sex and centrality

Model	Pseudo log likelihood	Deviance	df	∆χ²	∆df	*p*	AIC	BIC
**Symptoms of depression**								
No effect modification	-36,623	73,245	7				73,259	73,311
Effect modification by sex*	-36,390	72,781	10	465	3	< 0.001	72,801	72,875
Effect modification by urbanity	-36,620	73,239	13	6	6	0.408	73,265	73,361
**Depressive disorder**								
No effect modification	-4139	8277	4				8285	8315
Effect modification by sex*	-4033	8066	7	212	3	< 0.001	8080	8131
Effect modification by urbanity	-4129	8258	10	19	6	0.004	8278	8352
**Symptoms of conduct disorder**								
No effect modification	-22,603	45,206	7				45,220	45,272
Effect modification by sex*	-22,537	45,075	10	131	3	< 0.001	45,095	45,169
Effect modification by urbanity	-22,598	45,197	13	9	6	0.174	45,223	45,320
**Conduct problems**								
No effect modification	-3781	7562	4				7570	7600
Effect modification by sex*	-3737	7474	7	88	3	< 0.001	7488	7540
Effect modification by urbanity	-3773	7546	10	16	6	0.012	7566	7640
**Any alcohol use**								
No effect modification	-5567	11,135	4				11,143	11,172
Effect modification by sex	-5553	11,106	7	29	3	< 0.001	1120	11,172
Effect modification by urbanity*	-5527	11,055	16	80	12	< 0.001	11,075	11,149
**Risky alcohol use**								
No effect modification*	-3880	7759	4				7767	7797
Effect modification by sex	-3875	7750	7	9	3	0.027	7764	7816
Effect modification by urbanity	-3854	7707	10	52	6	< 0.001	7727	7802

*Model selected

The parameter estimates for the best fitting models are presented in Table 4. The Benjamini–Hochberg procedure [60] based on all the 36 *p*-values for these estimates, resulted in correcting the significance level from the commonly used *p* < 0.05 to *p* < 0.035.

**Table 4:** Growth model estimates for all study outcomes

	Symptoms of depression		Depressive disorder		Symptoms of conduct disorder		Conduct problems		Any alcohol use		Risky drinking	
	b (95% CI)	*p*	OR (95% CI)	*p*	b (95% CI)	*p*	OR (95% CI)	*p*	OR (95% CI)	*p*	OR (95% CI)	*p*
Intercept	5.43 (4.89, 5.96)	< 0.001	0.02 (0.01, 0.03)^1^	< 0.001	0.28 (0.18, 0.38)	< 0.001	0.01 (0.01, 0.02)^1^	< 0.001	0.00 (0.00, 0.00)^1^	< 0.001	0.00 (0.00, 0.00)^1^	< 0.001
Linear rate of change	1.90 (1.63, 2.17)	< 0.001	2.08 (1.68, 2.57)	< 0.001	0.11 (0.06, 0.15)	< 0.001	1.44 (1.09, 1.90)	0.011	8.10 (4.52, 14.53)	< 0.001	9.14 (6.66, 12.55)	< 0.001
Quadratic rate of change	-0.18 (-0.21, -0.14)	< 0.001	0.94 (0.60, 1.42)	< 0.001	-0.01 (-0.02, -0.00)	0.005	0.95 (0.91, 0.99)	0.008	0.95 (0.88, 1.03)	0.200	0.90 (0.87, 0.93)	< 0.001
Sex by initial status:												
Girls	Ref.		Ref.		Ref.		Ref.					
Boys	-1.92 (-2.56, -1.29)	< 0.001	0.18 (0.09, 0.36)	< 0.001	0.29 (0.13, 0.46)	< 0.001	2.67 (1.32, 5.41)	0.006				
Sex by linear rate of change:												
Female	Ref.		Ref.		Ref.		Ref.					
Male	-0.91 (-1.32, -0.50)	< 0.001	0.92 (0.60, 1.42)	0.716	0.10 (-0.00, 0.21)	0.059	1.02 (0.71, 1.48)	0.904				
Sex by quadratic rate of change:												
Girls	Ref.		Ref.		Ref.		Ref.					
Boys	0.10 (0.04, 0.16)	0.001	1.01 (0.95, 1.08)	0.674	-0.01 (-0.03, 0.00)	0.129	1.00 (0.95, 1.05)	0.928				
Centrality by initial status:												
High									0.81 (0.32, 2.04)	0.657		
Mid									Ref.			
Low									0.73 (0.25, 2.13)	0.562		
Centrality by linear rate of change:												
High									1.08 (0.61, 1.93)	0.790		
Mid									Ref.			
Low									2.83 (1.40, 5.71)	0.004		
Centrality by quadratic rate of change:												
High									1.03 (0.94, 1.13)	0.535		
Mid									Ref.			
Low									0.87 (0.79, 0.95)	0.004		

Confidence intervals and p-values are based on robust standard errors clustered at schools. The time metric in all models reflect chronological age, centered at age 13. ^1^Baseline odds conditional on zero random effects

**Fig. 1:**
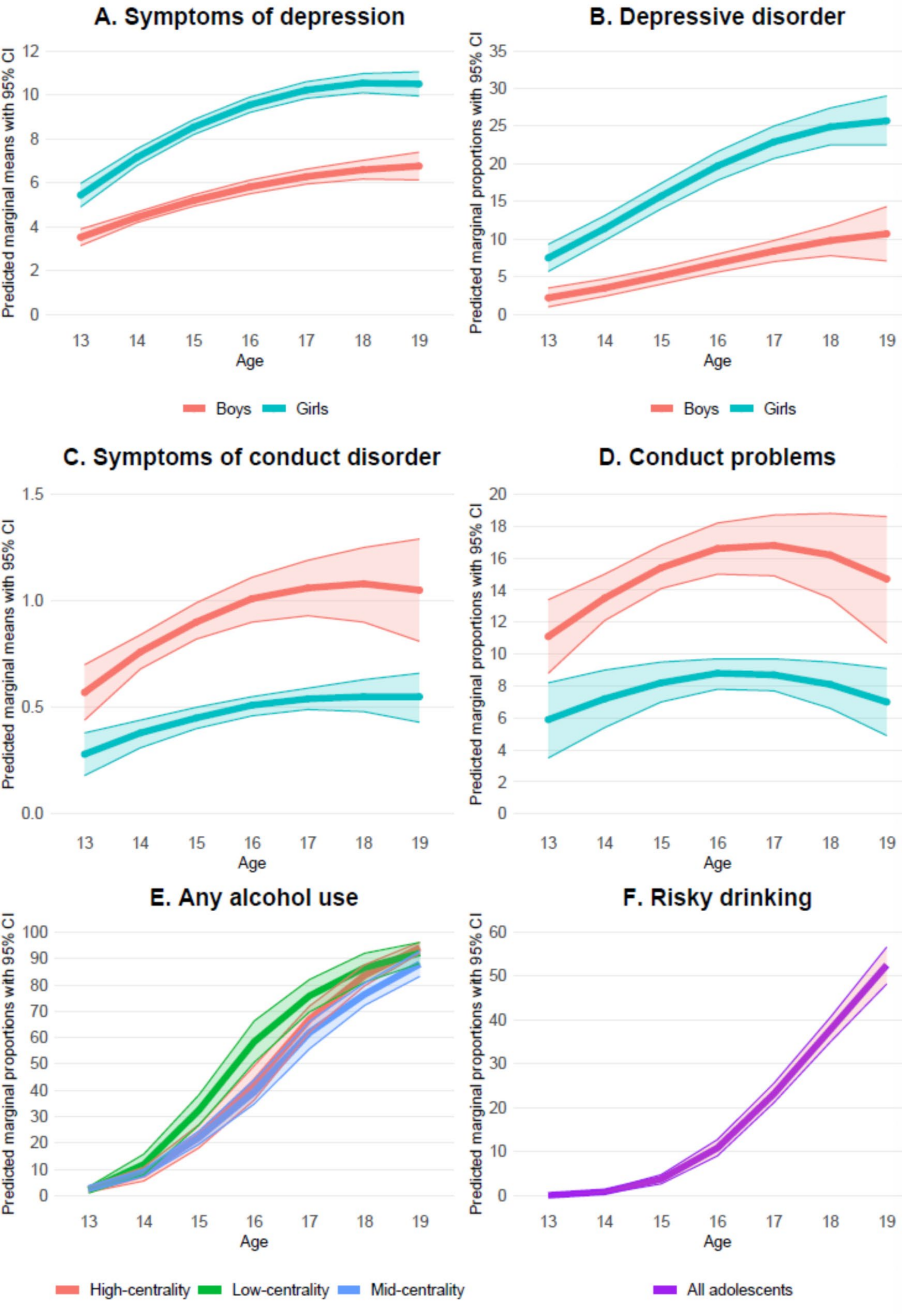
Developmental trajectories of depression, conduct problems, and alcohol use among Norwegian adolescents from age 13 to 19. Shaded areas denote 95% confidence intervals (CI)

The predicted marginal means and proportions from the multilevel models are presented in Supplementary Tables 2 and displayed graphically in Fig. 1.

### Symptoms of depression and rate of depressive disorder from age 13 to 19 years

The estimate for linear rate of change with age in Table 4 indicates that symptoms of depression increased for both sexes as they grew older (see panel A of Fig. 1). Girls had higher PHQ-9 scores at age 13, as indicated by the significant sex by initial status estimate. Girls also had greater increase over time as indicated by the estimate for sex by linear rate of change. The overall trajectories of depressive symptoms were curved, as indicated by a significant quadratic term, and for both sexes, the increase decelerated with age. Predicted marginal means indicated that girls peaked at 18 years, whereas boys had their highest score at age 19. There was significant between-person variance in both initial status (SD = 4.60, 95% CI: 4.25, 4.99) and in rate of change with age (SD = 1.05, 95% CI: 0.94, 1.17), demonstrating considerable heterogeneity in individual trajectories of depressive symptoms.

The results were similar for the dichotomous depressive disorder outcome. The predicted marginal means shown in panel B of Fig. 1, indicate that a larger proportion of girls had PHQ-9 symptom scores indicative of depressive disorder at age 13 compared to boys. The proportion of girls scoring within the depressive disorder range increased more rapidly throughout adolescence, such that by age 19, 26% of girls and about 11% of boys scored at or above the cut-off for depressive disorder. The absolute sex difference was greatest at age 18 and 19.

### Symptoms of conduct disorder and rate of conduct problems from age 13 to 19 years

Average symptoms of conduct disorder were low throughout adolescence. At age 13, boys scored higher than girls, as indicated by the significant sex by initial status estimate (see panel C of Fig. 1), and this sex difference persisted throughout adolescence. For both sexes, symptoms of conduct disorder increased overall, as indicated by the significant linear rate of change term. However, the increase levelled of as indicated by the significant quadratic term (see panel C of Fig. 1). There was significant between-person variance in both the initial status (SD = 0.57, 95% CI: 0.37, 0.90) and in rate of change with age (SD = 0.13, 95% CI: 0.07, 0.26), demonstrating considerable heterogeneity in individual trajectories of conduct problems.

A similar pattern was evident for prevalence of conduct problems (i.e., the proportion of our sample reporting two or more symptoms of conduct disorder) as shown in panel D of Fig. 1. The prevalence of conduct problems peaked at 17% at age 17 for boys and declined somewhat thereafter to age 19. The prevalence of conduct problems peaked at 9% at age 16 for girls and declined somewhat thereafter to age 19.

### Rates of any alcohol use and risky drinking from age 13 to 19 years

Evaluation of model fits for any alcohol use favoured different trajectories for adolescents from low, middle, and high centrality areas. As shown in the panel E of Fig. 1, less than 3% had consumed alcohol at age 13. Starting from age 14, there were increases in alcohol use with age in all three centrality groups. However, as indicated by the significant centrality by linear rate of change term, there were differences between the centrality groups. Alcohol use was more common among low centrality adolescents at age 15, 16, 17 and 18 compared to the middle-centrality adolescents, but it was more common only at age 16 compared to the high-centrality adolescents. At age 19 the three groups did not differ significantly.

For risky drinking, the moderation tests favoured a single trajectory across sex and centrality groups (panel F, Fig. 1). A very small percentage were risky drinkers at age 13, but as indicated by the significant linear and quadratic rate of change estimates, the percentage increased quadratically with age, such that by age 19, more than 50% were at or over the cut-off score for risky drinking.

## Discussion

The aim of this longitudinal study was to examine and describe normative developmental trajectories of depression, conduct problems, and alcohol use from ages 13 to 19 years among Norwegian post-millennium cohorts, and to explore whether these trajectories may differ for boys vs. girls, or for adolescents living in communities characterized by different levels of centrality. Consistent with prior studies, we observed significant increases in all outcomes across adolescence, with depression being more pronounced among girls and conduct problems being more pronounced among boys [61–6, 9, 11–14]. Consistent with the emerging evidence for the narrowing gender gap, we observed no meaningful differences in alcohol use between boys and girls. Nor did we observe any meaningful differences in these developmental trajectories as a function of centrality.

Specifically, both the self-reported symptoms of depression and the corresponding prevalence of depressive disorder increased in our sample during adolescence, but the increase was steeper in early than in late adolescence. In our study, the proportion of adolescents reporting symptom levels indicative of depressive disorder was highest at age 19. In line with previous findings [61–34, 31], both the initial levels and the increases over time were greater for girls than for boys. Sex differences in depressive symptoms might in part be explained by differences in hormonal changes and brain development that make girls more sensitive to the effects of stress [62, 63], and social-emotional differences [64].

Conduct problems also increased in early adolescence but levelled off and declined somewhat in later adolescence. In accordance with previous studies [6, 7, 9, 65, 66], both the number of conduct disorder symptoms and the proportion of participants scoring above our cut-off for conduct problems were greater for boys than for girls. In our study, the average number of symptoms of conduct disorder was low for both sexes, echoing previous results from Norway [65, 67].

Also in line with previous studies [10–14], the prevalence of alcohol use and risky drinking among adolescents from our sample were low in early adolescence, but both increased rapidly from age 14 and the increase accelerated with age. We did not observe notable sex differences in alcohol use. This is consistent with data from other Western European countries, however greater rates of risky drinking among boys have been reported for some Eastern European countries [68]. In our study, the estimated prevalence of risky drinking was upwards of 50% at age 19. This is concerning, especially considering that we applied a rather conservative cut-off point for risky drinking [56].

We also examined putative differences in developmental trajectories of depression, conduct problems, and alcohol use between adolescents as a function of the centrality of their place of residence– an important proxy for several socio-economic indicators and structural determinants of health [38, 39, 69]. We found no notable differences in adolescents’ depression trajectories based on their locality characteristics. Our findings diverge from previous Norwegian research documenting stronger burden of depression symptoms in urban locations in limited geographic areas and in cohorts born prior to 2000 [70]. Our results are however consistent with a more recent Norwegian study reporting negligible differences in depressive symptoms according to centrality [45]. Our study also did not provide any evidence for differences in conduct problems as a function of centrality, echoing the results of a recent study from Finland [71]. The only notable difference according to centrality was observed for any alcohol use, where the prevalence was higher in mid-adolescence among adolescents from less central communities. These findings are somewhat similar to results from older studies documenting higher rates of early alcohol initiation among Danish adolescents from rural communities [72], but are divergent from studies documenting higher drinking frequency among Finnish adolescent girls (but not boys) from urban communities [71]. Importantly, in our study this pattern was evident only for *any* drinking; we found no differences in *risky* drinking, which was not examined in previous studies. Risky drinking can have more serious consequences than more moderate drinking, therefore our results indicate that prioritizing low-centrality communities for alcohol prevention might not be required.

### Implications

The prevalence of depression and risky drinking in our sample was considerable, underscoring the need for early prevention and treatment of these specific issues. As girls appear to be affected by depression both more severely in terms of overall symptomatology and in greater numbers, a stronger focus on prevention and treatment for girls might be beneficial. For instance, targeted prevention programs such as Interpersonal Psychotherapy Adolescent Skills Training [73], and services for adolescents such as Headspace [74] could focus more on recruiting girls and being more relevant for girls in particular. Even though conduct problems were uncommon in this sample, some adolescents did engage in misconduct such as destruction, stealing, and fighting. Preventing long term consequences by targeting this high-risk group, for instance by Multisystemic therapy [75], may be more appropriate than preventive efforts aimed at the general adolescent population.

Our results further indicate that depression and conduct problems may be present before age 13, suggesting that the related prevention efforts in Norway might be more meaningful if implemented in primary rather than in secondary school. In contrast, as we observed sharp increases in alcohol use primarily after age 14, our results suggest that implementation of substance use prevention efforts during lower secondary school may be optimal. This is supported by additional evidence that young Norwegians typically hold negative alcohol expectancies in early adolescence, but that these tend to become accompanied by positive expectancies later in adolescence [76]. Finally, we only found small centrality effects, implying little need for community-tailored preventive efforts in Norway. Indeed, as Norway is a high-income country characterized by a generous welfare state committed to reduction of social inequalities and poor health [77], there might be less inter-municipality variation in living conditions compared to other countries.

### Strengths and limitations

We examined data from a large, geographically and socio-economically heterogeneous, and nationwide cohort of post-millennial adolescents who completed five annual assessments thus enabling modelling of complex, non-linear developmental trajectories across adolescence. Measures of depression, conduct problems, and alcohol use were based on well-established instruments with clinical relevance and meaningful cut-off criteria. Centrality was determined via an official registry by employing a new and improved centrality index [39].

Some study limitations should also be noted. All three outcomes were self-reported, which can lead to recall bias, socially desirable responding, and measurement error [78]. Several assessments took place during the COVID-19 pandemic, however previous studies with this sample indicate little impact of the pandemic on the studied outcomes [79, 80]. We found higher attrition for older adolescents and adolescents with more conduct problems; hence observations of the outcome variables were most likely not missing completely at random (MCAR). However, data from all the individuals in the dataset (including individuals with missing observations at some assessment timepoints) were included in the mixed models, which can yield unbiased estimates under the missing at random (MAR) assumption [59].

Application of the Benjamini-Hochberg procedure yielded a corrected significance level of *p* < 0.035 for the multilevel-modelling regression coefficients. Had we employed the more stringent Bonferroni correction, the adjusted alpha level would have been *p* < 0.001. Under this stricter criterion, our analysis would have supported a linear rather than quadratic rate of change for symptoms of conduct disorder, and no significant change over time and no observed sex differences for conduct problems. However, we did not use the Bonferroni correction because it can inflate the type II error rate [81, 82].

## Conclusion

Consistent with research on previous cohorts of adolescents, Norwegian adolescents born after 2000 have increasing average levels of depression and conduct problems during adolescence and increasing rates of depressive disorder, conduct problems, alcohol use and risky drinking. Depression was more prevalent among girls, whereas boys faced greater challenges with conduct problems. Interestingly, the development of risky drinking showed a similar trajectory for both sexes. The heavy burden on adolescents caused by depression, conduct problems and risky drinking highlights the need for prevention and treatment. Our results suggest that prevention programs can be introduced at the same early age in rural and urban locations because of similar developmental trajectories.

## Supplementary Information

Supplementary Material 1.

## Data Availability

No datasets were generated or analysed during the current study.
