# Supplementary material for: Depressive symptoms, conduct problems and alcohol use from age 13 to 19 in Norway: evidence from the MyLife longitudinal study
Source: Child Adolesc Psychiatry Ment Health. 2024 Oct 9;18:127. doi: 10.1186/s13034-024-00824-x (PMC11465494; doi:10.1186/s13034-024-00824-x)
Supplement: Supplementary file 1 — Supplementary Material 1. [file 13034_2024_824_MOESM1_ESM.doc]

Supplementary material for:

Article title: Depressive symptoms, conduct problems and alcohol use from age 13 to 19 in Norway: Evidence from the MyLife longitudinal study

Journal: European Child & Adolescent Psychiatry

Authors: Geir Scott Brunborg^1,2^, Lasse Bang^1^, Jens Christoffer Skogen^3,4,5^, Jasmina Burdzovic Andreas^6,7^

Affiliations: ^1^Department of Child Health and Development, Norwegian Institute of Public Health, Oslo, Norway

^2^[Department of Clinical Neuroscience](https://staff.ki.se/orgid/5208), Karolinska Institutet, Stockholm, Sweden

^3^Department of Health Promotion, Norwegian Institute of Public Health, Bergen, Norway

^4^Centre for Evaluation of Public Health Measures, Norwegian Institute of Public Health, Oslo, Norway

^5^Alcohol and Drug Research Western Norway, Stavanger University Hospital, Stavanger, Norway

^6^Department of Alcohol, Tobacco and Drugs, Norwegian Institute of Public Health, Oslo, Norway

^7^Department of Psychology, University of Oslo, Oslo, Norway

Correspondence concerning this article should be addressed to Geir Scott Brunborg, Norwegian Institute of Public Health, PO Box 222-Skøyen, N-0213 Oslo, Norway. Email: [geir.brunborg@fhi.no](mailto:geir.brunborg@fhi.no)

| Supplementary table 1. Response frequencies (%) for specific conduct problems by sex and age. | | | | | | | |  |  |  |
| --- | --- | --- | --- | --- | --- | --- | --- | --- | --- | --- |
|  |  |  | | | |  |  |  |  |  |
|  |  | Girls | | | |  | Boys | | | |
| Questionnaire item | Age | "Never" | "1 or 2 times" | "3 or 4 times" | "5 or more times" |  | "Never" | "1 or 2 times" | "3 or 4 times" | "5 or more times" |
| "Did you intentionally destroy something that did not belong to you?" | 13 | 91.8 | 7.1 | 1.1 | 0.0 |  | 83.6 | 14.3 | 1.1 | 1.1 |
|  | 14 | 90.2 | 8.9 | 0.7 | 0.1 |  | 82.5 | 14.4 | 1.8 | 1.4 |
|  | 15 | 92.3 | 6.6 | 0.6 | 0.5 |  | 82.2 | 14.0 | 1.8 | 2.0 |
|  | 16 | 93.3 | 5.9 | 0.6 | 0.2 |  | 83.3 | 12.6 | 2.0 | 2.0 |
|  | 17 | 94.6 | 4.5 | 0.7 | 0.2 |  | 85.3 | 9.8 | 2.8 | 2.1 |
|  | 18 | 96.1 | 3.1 | 0.5 | 0.3 |  | 89.2 | 8.0 | 1.7 | 1.2 |
|  | 19 | 97.3 | 2.2 | 0.4 | 0.0 |  | 94.0 | 4.5 | 1.1 | 0.4 |
| "Did you get into a serious fight?" | 13 | 98.1 | 1.9 | 0.0 | 0.0 |  | 89.0 | 8.9 | 1.4 | 0.7 |
|  | 14 | 97.3 | 2.2 | 0.2 | 0.3 |  | 90.0 | 8.4 | 0.8 | 0.8 |
|  | 15 | 97.4 | 2.1 | 0.3 | 0.2 |  | 91.6 | 6.3 | 0.9 | 1.2 |
|  | 16 | 97.6 | 2.0 | 0.3 | 0.1 |  | 91.8 | 6.0 | 1.3 | 0.9 |
|  | 17 | 98.2 | 1.5 | 0.0 | 0.1 |  | 92.7 | 4.8 | 1.6 | 0.9 |
|  | 18 | 97.9 | 1.6 | 0.4 | 0.1 |  | 91.7 | 5.9 | 1.9 | 0.5 |
|  | 19 | 98.4 | 1.2 | 0.4 | 0.0 |  | 94.4 | 4.5 | 0.7 | 0.4 |
| "Did you stay out all night without your parents knowing where you were?" | 13 | 97.3 | 2.2 | 0.2 | 0.3 |  | 97.2 | 1.1 | 1.1 | 0.7 |
|  | 14 | 96.8 | 2.5 | 0.4 | 0.3 |  | 94.4 | 4.3 | 0.5 | 0.7 |
|  | 15 | 93.4 | 5.3 | 0.9 | 0.5 |  | 92.2 | 5.2 | 1.2 | 1.4 |
|  | 16 | 89.5 | 7.5 | 1.8 | 1.2 |  | 88.6 | 7.9 | 1.5 | 1.9 |
|  | 17 | 84.8 | 10.0 | 3.1 | 2.1 |  | 84.9 | 8.1 | 3.5 | 3.4 |
|  | 18 | 82.2 | 11.2 | 3.8 | 2.8 |  | 82.0 | 12.6 | 3.0 | 2.4 |
|  | 19 | 80.8 | 12.5 | 2.4 | 4.3 |  | 73.1 | 15.2 | 6.7 | 4.9 |
| "Did you steal something valuable from others?" | 13 | 99.2 | 0.5 | 0.0 | 0.3 |  | 98.2 | 1.4 | 0.4 | 0.0 |
|  | 14 | 97.5 | 1.8 | 0.6 | 0.1 |  | 96.8 | 2.4 | 0.3 | 0.5 |
|  | 15 | 98.3 | 1.2 | 0.1 | 0.3 |  | 96.8 | 1.6 | 0.7 | 0.9 |
|  | 16 | 98.1 | 1.1 | 0.4 | 0.3 |  | 96.0 | 2.5 | 0.9 | 0.6 |
|  | 17 | 97.7 | 1.7 | 0.2 | 0.4 |  | 94.2 | 3.6 | 1.4 | 0.8 |
|  | 18 | 96.8 | 1.7 | 0.9 | 0.5 |  | 94.4 | 4.1 | 0.8 | 0.7 |
|  | 19 | 99.0 | 0.6 | 0.4 | 0.0 |  | 95.5 | 2.2 | 1.9 | 0.4 |
| "Did you behave noisily, rowdily, or disruptively in a public place?" | 13 | 93.4 | 4.7 | 0.8 | 1.1 |  | 85.1 | 11.3 | 1.8 | 1.8 |
|  | 14 | 90.3 | 7.4 | 1.5 | 0.8 |  | 81.9 | 12.3 | 2.2 | 3.7 |
|  | 15 | 87.3 | 9.8 | 1.4 | 1.5 |  | 81.4 | 12.9 | 2.9 | 2.9 |
|  | 16 | 89.2 | 8.4 | 1.2 | 1.1 |  | 80.4 | 13.2 | 3.2 | 3.2 |
|  | 17 | 89.4 | 8.1 | 1.8 | 0.7 |  | 82.3 | 10.9 | 3.7 | 3.1 |
|  | 18 | 92.1 | 6.0 | 1.5 | 0.3 |  | 84.7 | 11.2 | 2.5 | 1.5 |
|  | 19 | 92.6 | 5.5 | 1.4 | 0.4 |  | 83.6 | 12.3 | 3.3 | 0.7 |
| "Did you bully others?" | 13 | 98.3 | 1.7 | 0.0 | 0.0 |  | 98.6 | 1.1 | 0.0 | 0.4 |
|  | 14 | 96.4 | 3.4 | 0.1 | 0.1 |  | 94.6 | 3.8 | 0.7 | 0.9 |
|  | 15 | 95.9 | 3.2 | 0.1 | 0.3 |  | 93.9 | 4.2 | 0.6 | 1.3 |
|  | 16 | 96.7 | 2.9 | 0.3 | 0.0 |  | 92.3 | 5.1 | 1.2 | 1.3 |
|  | 17 | 96.6 | 2.7 | 0.4 | 0.4 |  | 91.7 | 4.8 | 1.6 | 1.8 |
|  | 18 | 97.8 | 1.5 | 0.6 | 0.1 |  | 92.2 | 4.8 | 2.0 | 1.0 |
|  | 19 | 97.3 | 1.8 | 0.6 | 0.2 |  | 95.5 | 3.3 | 0.4 | 0.7 |

| Supplementary table 2. Predicted marginal means and percentages with lower and upper 95% confidence intervals. | | | | | | |  |
| --- | --- | --- | --- | --- | --- | --- | --- |
| Outcome | Age | | | | | | |
|  | 13 | 14 | 15 | 16 | 17 | 18 | 19 |
| **Symptoms of depression, mean** |  |  |  |  |  |  |  |
| Girls (lower) | 4.89 | 6.77 | 8.18 | 9.19 | 9.83 | 10.09 | 9.94 |
| Girls | 5.43 | 7.15 | 8.52 | 9.54 | 10.21 | 10.53 | 10.49 |
| Girls (upper) | 5.96 | 7.54 | 8.86 | 9.89 | 10.59 | 10.96 | 11.04 |
| Boys (lower | 3.13 | 4.18 | 4.92 | 5.49 | 5.93 | 6.16 | 6.12 |
| Boys | 3.51 | 4.42 | 5.18 | 5.80 | 6.27 | 6.58 | 6.75 |
| Boys (lower) | 3.88 | 4.66 | 5.44 | 6.11 | 6.61 | 7.01 | 7.38 |
| **Depressive disorder, %** |  |  |  |  |  |  |  |
| Girls (lower) | 5.7 | 9.8 | 14.0 | 17.8 | 20.7 | 22.5 | 22.5 |
| Girls | 7.5 | 11.4 | 15.7 | 19.7 | 22.9 | 24.9 | 25.7 |
| Girls (upper) | 9.3 | 13.1 | 17.4 | 21.6 | 25.0 | 27.4 | 29.0 |
| Boys (lower | 1.0 | 2.4 | 4.0 | 5.6 | 7.0 | 7.8 | 7.1 |
| Boys | 2.2 | 3.5 | 5.1 | 6.8 | 8.4 | 9.8 | 10.7 |
| Boys (lower) | 3.5 | 4.7 | 6.2 | 8.0 | 9.8 | 11.8 | 14.3 |
| **Symptoms of conduct disorder, mean** |  |  |  |  |  |  |  |
| Girls (lower) | 0.18 | 0.31 | 0.40 | 0.46 | 0.49 | 0.48 | 0.43 |
| Girls | 0.28 | 0.38 | 0.45 | 0.51 | 0.54 | 0.55 | 0.55 |
| Girls (upper) | 0.38 | 0.44 | 0.50 | 0.55 | 0.59 | 0.63 | 0.66 |
| Boys (lower | 0.44 | 0.68 | 0.82 | 0.90 | 0.93 | 0.90 | 0.81 |
| Boys | 0.57 | 0.76 | 0.90 | 1.01 | 1.06 | 1.08 | 1.05 |
| Boys (lower) | 0.70 | 0.84 | 0.99 | 1.11 | 1.19 | 1.25 | 1.29 |
| **Conduct problems, %** |  |  |  |  |  |  |  |
| Girls (lower) | 3.5 | 5.4 | 7.0 | 7.8 | 7.7 | 6.6 | 4.9 |
| Girls | 5.9 | 7.2 | 8.2 | 8.8 | 8.7 | 8.1 | 7.0 |
| Girls (upper) | 8.2 | 9.0 | 9.5 | 9.7 | 9.7 | 9.5 | 9.1 |
| Boys (lower | 8.8 | 12.1 | 14.1 | 15.0 | 14.9 | 13.5 | 10.7 |
| Boys | 11.1 | 13.5 | 15.4 | 16.6 | 16.8 | 16.2 | 14.7 |
| Boys (upper) | 13.4 | 15.0 | 16.8 | 18.2 | 18.7 | 18.8 | 18.6 |
| **Any alcohol use, %** |  |  |  |  |  |  |  |
| High-centrality (lower) | 1.4 | 5.6 | 18.2 | 36.3 | 62.0 | 79.6 | 92.0 |
| High-centrality | 2.4 | 8.2 | 22.6 | 42.8 | 66.9 | 83.6 | 94.1 |
| High-centrality (upper) | 3.4 | 10.8 | 27.0 | 49.3 | 71.9 | 87.6 | 96.2 |
| Mid-centrality (lower) | 1.7 | 7.3 | 19.6 | 34.8 | 55.7 | 72.2 | 83.2 |
| Mid-centrality | 2.7 | 8.7 | 22.1 | 39.4 | 61.9 | 76.4 | 87.9 |
| Mid-centrality (upper) | 3.7 | 10.0 | 24.6 | 44.0 | 66.1 | 80.7 | 92.7 |
| Low-centrality (lower) | 1.0 | 8.0 | 26.8 | 50.6 | 69.7 | 81.0 | 88.1 |
| Low-centrality | 2.2 | 11.9 | 32.5 | 58.4 | 75.8 | 86.5 | 92.1 |
| Low-centrality (upper) | 3.4 | 15.8 | 38.3 | 66.3 | 82.0 | 92.0 | 96.1 |
| **Risky drinking, %** |  |  |  |  |  |  |  |
| All (lower) | 0.0 | 0.5 | 2.7 | 9.1 | 21.2 | 35.0 | 48.2 |
| All | 0.1 | 0.9 | 3.8 | 10.9 | 23.2 | 37.8 | 52.5 |
| All (upper) | 0.2 | 1.3 | 4.8 | 12.8 | 25.6 | 40.6 | 56.6 |
